# Prospective cohort study using the breast cancer spheroid model as a predictor for response to neoadjuvant therapy – the SpheroNEO study

**DOI:** 10.1186/s12885-015-1491-7

**Published:** 2015-07-15

**Authors:** Kathrin Halfter, Nina Ditsch, Hans-Christian Kolberg, Holger Fischer, Tanja Hauzenberger, Franz Edler von Koch, Ingo Bauerfeind, Gunter von Minckwitz, Ilona Funke, Alexander Crispin, Barbara Mayer, Behalf of the SpheroNEO Study Group

**Affiliations:** 1 Department of General, Visceral, Transplantation, Vascular and Thoraic Surgery, Hospital of the University of Munich, Munich, Germany; 2 Department of Obstetrics and Gynecology, Ludwig-Maximilians-University of Munich, Munich, Germany; 3 Marienhospital Bottrop, Bottrop, Germany; 4 Evangelische Kliniken Gelsenkirchen, Gelsenkirchen, Germany; 5 Klinikum St. Marien Amberg, Amberg, Germany; 6 Department of Obstetrics and Gynecology, Klinikum Dritter Orden, Munich, Germany; 7 Klinikum Landshut, Landshut, Germany; 8 GBG Forschungs GmbH, Neu-Isenburg and University Women’s Hospital Frankfurt, Frankfurt, Germany; 9 SpheroTec GmbH, Martinsried, Germany; 10 IBE LMU, Department of Obstetrics and Gynecology, Technical University of Munich, Klinikum Starnberg, Leopoldina Krankenhaus der Stadt Schweinfurt, Markus Krankenhaus Frankfurt, Klinikum Nürnberg, Städtisches Klinkum Karlsruhe, Klinikum Harlaching, Munich, Germany

**Keywords:** Breast cancer, Neoadjuvant chemotherapy, Predicting treatment outcome, Cellular response to anticancer drugs, Personalized medicine, in vitro diagnostics

## Abstract

**Background:**

Aim of this prospective study was to predict response to neoadjuvant therapy in breast cancer patients using an *in vitro* breast cancer spheroid model.

**Methods:**

Three-dimensional spheroids were directly generated from fresh breast tumor biopsies of 78 patients eligible for neoadjuvant therapy. Cell survival was measured after *in vitro* exposure to the equivalent therapeutic agents in the breast cancer spheroid model. Treatment results *in vitro* were correlated with pathological complete response (pCR, i.e. ypT0 ypN0) determined at surgery.

**Results:**

A mean cell survival of 21.8 % was found in the breast cancer spheroid model for 22 patients with pCR versus 63.8 % in 56 patients without pCR (*P* = .001). The area under the receiver operator characteristic curve to predict pCR was 0.86 (95 % CI: 0.77 to 0.96) for cell survival *in vitro* compared to 0.80 (95 % CI: 0.70 to 0.90) for a combined model of conventional factors (hormone- and HER2 receptor, and age). A cutoff at 35 % cell survival for the spheroid model was proposed. Out of the 32 patients with values below this threshold, 21 patients (65.6 %) and one patient (2.2 %) with a cell survival greater than 35 % achieved pCR respectively; (sensitivity 95.5 % (95 % CI: 0.86 to 1.00); specificity 80.4 % (95 % CI: 0.70 to 0.91)). Extent of residual disease positively correlated with increased cell survival (*P* = .021).

**Conclusion:**

The breast cancer spheroid model proved to be a highly sensitive and specific predictor for pCR after neoadjuvant chemotherapy in breast cancer patients.

**Electronic supplementary material:**

The online version of this article (doi:10.1186/s12885-015-1491-7) contains supplementary material, which is available to authorized users.

## Background

Multiple studies in breast cancer have shown that pathological complete response (pCR) serves as a reliable surrogate marker for progression-free survival, as well as overall survival [1–6]. Despite all efforts, the rate of pathological complete response (pCR) following neoadjuvant therapy for primary breast cancer remains low at an average rate of 20-30 % [1, 7]. Efforts to maximize the outcome of the standard neoadjuvant treatment have been tested in numerous clinical trials, with variations in dosing such as dose-dense or dose-intensified regimen [8], or order of application of single-agent and combination treatment regimen [9, 10]. In addition, established cytostatic agents or new drugs targeting HER2, angiogenesis, or mammalian target of Rapamycin (mTOR) have been combined to novel therapy strategies [11–16]. The application of targeted therapy in combination with a taxane/anthracycine-based regimen with the addition of carboplatin resulted in an increase of the rate of pCR by 16.3 % in triple negative breast cancer patients [17]. Other trials have not yielded such promising results or the data obtained was inconclusive and did not warrant changes in guideline recommendations. The addition of capecitabine to a standard anthracycline/taxane-based regimen [14], the sequence of paclitaxel and 5-FU/anthracycline/cyclophosphamide combination therapy [10], or the treatment of early non-responders with the mTOR-inhibitor everolimus given simultaneously with paclitaxel [15] did not show any improvement of pCR. Encouraging developments have been made regarding single and dual agent anti-HER2 blockade. This treatment option raised the rate of pCR among women with HER2-positive tumors up to 66.7 % [18–20]. Unfortunately, among the HER2 positive patient population only a limited proportion show a treatment response to HER2 inhibition [21, 22]. In addition, only 20 % of all tumors diagnosed are positive for this targetable biomarker [23], leaving the majority of women with no further treatment options aside from the standard chemotherapy treatment. So far, no biomarker has been accepted for routine use to accurately predict treatment outcome to chemo- and/or anti-HER2-therapy.

Currently predictive diagnostic tests are available that stratify patients to an individual treatment regimen using a genetic or cellular approach. Assays based on the genetic analysis of the tumor tissue make up the largest proportion; some of these tests have already been validated for specified subgroups in prospective trials [24–26]. However, no data that would indicate the most effective treatment option is provided even though numerous treatment options in breast cancer treatment are available [24, 27]. Cell-based chemosensitivity and chemoresistance assays, such as single-cell suspension and cell monolayer assays [28–31], are currently not sufficiently validated for clinical implementation [32]. Contrary to the 2-Dimensional (2D) cell culture models, 3-Dimensional (3D) spheroid models reflect the tumor biology and tumor microenvironment much more accurately [33–38]. The spheroid-based assay proposed herein, was assessed as a diagnostic tool to aid in the therapeutic decision-making. The objective of the SpheroNEO study was to test whether *in vitro* treatment results obtained in the breast cancer spheroid model are associated with treatment outcome in primary breast cancer patients undergoing neoadjuvant therapy.

## Methods

Starting from October 2009 until September 2012, 202 patients from 13 breast cancer centers in Germany were enrolled in the SpheroNEO study. Written consent for the trial was given by all applicable ethics committees. An informed consent was obtained from all eligible patients (Additional file 1). Patients 18 years or older were eligible if a clinically confirmed case of invasive breast cancer had been diagnosed and the use of neoadjuvant chemotherapy was recommended. Patients with a previous diagnosis and/or treatment of a malignant disease, as well as patients with metastatic disease were excluded.

### Study design

The SpheroNEO study was designed as a prospective, non-interventional cohort study. Tumor tissue from core needle biopsies was obtained simultaneously for the SpheroNEO study and histopathological diagnosis. Drugs tested in the breast cancer spheroid model were recommended by the treating physicians of the breast cancer center at the time of the biopsy procedure. Results obtained in the breast cancer spheroid model had no impact on the treatment decision for the individual patient, and treating physicians were blinded to the results of the laboratory test. A comparison of the therapeutic response *in vitro* and clinical treatment outcome documented in the pathological report was performed after all patients had completed the neoadjuvant treatment followed by surgery. Pathological complete response was defined as no vital tumor in breast or axilla (pCR, i.e. ypT0 ypN0) determined at surgery following the completion of chemotherapy.

### Breast cancer spheroid model

Fresh tumor biopsy samples were collected in freshly prepared culture medium containing DMEM/F12-medium (PAN), 10 % fetal calf serum (PAN), 2x MEM non-essential amino acid solution (PAN), 2x MEM vitamin solution NEAA, as well as a mixture of antibiotic/antifungal compounds (0.26 μM Amphotericin B, Ampicillin 0.14 mM, Ciprofloxacin 7.54 μM) and shipped from participating breast cancer centers to an external laboratory. All laboratory procedures and tests were performed according to standardized, quality-controlled handling procedures. The tumor samples underwent mechanical and enzymatic digestion using an enzyme cocktail containing Liberase TM, which consisted of a mixture of collagenases and neutral protease enzymes (Roche, Penzberg Germany). After determination of cell viability using the trypan-blue exclusion test, the single cell suspension was directly processed into breast cancer spheroids using a modified liquid overlay technique [39, 40]. No red blood cell lysis was performed. A training cohort of 14 breast tumor biopsies was tested prior to study start, to optimize the assay protocol. This was necessary since the previously described liquid overlay method used RPMI culture medium for basic cell line culture, this was replaced by DMEM/F12 to better accommodate primary tumor cells. The cell isolation procedure was also supplemented to include additional washing steps to filter out cellular debris. For this purpose the use of a cell strainer was added to the cell isolation procedure as well.

The spheroids were cultured for 48 h at 37 °C and 5 % CO_2_ and treated with the recommended combination of cytostatic agents using the peak plasma concentration of each drug (ppc), see Table 1 for the utilized concentrations and solvent controls. Spheroid formation was verified and grade of compaction was documented using bright field microscopy prior to drug treatment. Medium was not changed during any time during the spheroid culture. The duration of the drug treatment was 96 h after which the treatment efficacy was assessed using a standard assay measuring metabolic activity (Promega, Mannheim Germany) to quantify cell survival *in vitro*. Mean cell survival was expressed as percent residual metabolic activity of the respective solvent controls. Laboratory test results were available after eight days.

**Table 1:** Cytostatic compounds used in the breast cancer spheroid model

Name	PPC conzentration [μg/ml]	Literature source
Carboplatin	40.843	Go, Adjei, 1999 [53]
Cyclophosphamid	41.000	Egorin et al, 1989 [54]
Docetaxel	2.180	Baker et al, 2004 [55]; Bruno et al, 1998 [56]
Doxorubicin	1.640	Brana et al, 2014 [57]
Epirubicin	1.005	Reviewed in Fujimoto, 2007 [58]
Fluorouracil (5-FU)	100.000	Reviewed in Fujimoto, 2007 [58]
Paclitaxel	1.530	Gianni et al, 1995 [59]
Trastuzumab	88.000	Leyland-Jones, 2001 [60]

### Statistics

Sample size was estimated based on a 95 % true-positive rate (TPR) and a true negative rate (TNR) of 90 % for the breast cancer spheroid model. If 20 % of the patients achieved pCR, a sample size of 70 was regarded as sufficient to estimate both TPR and TNR with a lower limit of the 95 % Clopper-Pearson interval greater than 66 %.

All results are reported from the intention-to-treat analysis. Clinical and laboratory data was described separately for patients with and without pCR using appropriate measures of location and dispersion. Analyses of the discriminatory power of the spheroid model and traditional risk factors were based on receiver operating characteristic (ROC) curves and the corresponding c statistics (areas under the ROC curves, AUC) calculated using the SAS LOGISTIC procedure. Additional logistic models were fit to estimate the role of the assay results in the context of traditional risk factors, using forward selection based on Wald tests on an alpha level of 5 %. Since this frequently lead to a disproportion between the number of the covariates and the sample size, the analysis was replicated using penalized (lasso) logistic regression in the R package “penalized”. DeLong 95 % confidence intervals for the c statistics from the lasso regression models were calculated using the R package ‘pROC’. The 95th percentile of the residual activity in women with pCR was used as cut-off and the resulting sensitivity and specificity and their 95 % confidence limits are reported, calculation of the Youden Index resulted in the same value. Laboratory data was correlated with pCR using Pearson’s chi-square or Fisher’s exact tests for categorical factors and t-tests for numerical variables. The correlation between cell survival *in vitro* after treatment and the size of residual tumor in the breast (ypT) after neoadjuvant therapy was quantified using Spearman's rank correlation coefficient. All hypotheses tested were two-sided on an alpha level of 5 %. Analyses were performed using the Statistical Analysis System SAS, version 9.2 for Linux (SAS Institute, Cary, NC), as well as R version 2.12.2 for Windows (The R Foundation for Statistical Computing).

## Results

**Fig. 1:**
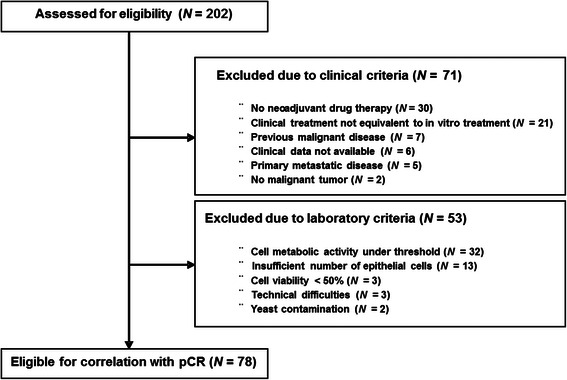
Study flowchart depicting the screening process of the patients in the SpheroNEO cohort

A total of 202 patients were enrolled in the SpheroNEO study. Figure 1 shows in detail the exclusion criteria for the screened patients. The main clinical reasons for exclusion were both related to choice of treatment. This was due to the fact that at the time of biopsy the results from the staging examinations were not yet available, and a definitive treatment decision had not been made. A total of 30 patients did not receive neoadjuvant treatment as initially planned, instead undergoing primary surgery followed by adjuvant treatment. The second main reason for exclusion (*n* = 21) was the discrepancy between clinical therapy and *in vitro* treatment, due to results from final staging examinations or patient preferences.

As to the laboratory criteria, the main reason for exclusion was the limited amount of cells isolated from the biopsies. Due to the limited number of available cells no additional assays were possible. This resulted in an insufficient number of isolated cells (calculated minimum of 2841 cells required; *n* = 13) or a metabolic activity below threshold (*n* = 32). The mean duration from biopsy procedure to the start of tissue preparation was 26.4 h (range 1.0 – 162.8 h). A breast cancer spheroid assay was considered successful when the minimal metabolic of the untreated controls measured at least 107.5 counts per second (cps). This threshold represented twice the mean luminescence for the solvent controls without cells. Results show that a minimum of four biopsy cylinders with a mean total weight of 89.6 mg (range 10.5 – 353.4 mg), were required to test a minimum of three treatment combinations. The final analysis included 78 patients, who qualified according to both clinic and laboratory criteria.

### Baseline characteristics of the SpheroNEO study cohort

Baseline characteristics are shown in Table 2. Age, clinical tumor stage, tumor histology, and receptor status were representative compared to previously published larger cohorts [6, 41]. All except two patients received taxane-based chemotherapy combined with either anthracycline or carboplatin. All patients with HER2-positive tumors received trastuzumab-based therapy (20.5 %). Patient tumors were most frequently invasive ductal in their histology (61 out of 78; 78.2 %) with a median diameter of 2-5 cm (cT2; 45 out of 77; 58.4 %). Regarding tumor biology, 64.5 % (49 out of 76) and 53.2 % (42 out of 77) were positive for estrogen- and progesterone receptor respectively.

**Table 2:** Baseline Characteristics and pCR Rates of the SpheroNEO cohort

			pCR				
	All Patients		Yes		No		
Characteristics	n	%	n	%	n	%	*P*
All Patients	78	100	22	28.2	56	71.8	
Age at diagnosis, years							.207
≤50	43	55.1	15	34.9	28	65.1	
>50	35	44.9	7	20.0	28	80.0	
Mean	51 21 - 78		46 21 - 65		53 25 - 78		.029
Range							
Tumor stage							.779
cT1/T2	56	72.7	16	28.6	40	71.4	
cT3/4	21	27.3	5	23.8	16	76.2	
Not documented	1	-					
Nodal status							1.000
cN+	42	54.5	12	28.6	30	71.4	
cN-	35	45.5	10	28.6	25	71.4	
Not documented	1	-					
Grading							.123
G1/2	41	53.9	8	19.5	33	80.5	
G3	35	46.1	13	37.1	22	62.9	
Not documented	2	-					
Histologic type							.449
Invasive ductal/other	70	89.7	21	30.0	49	70.0	
Invasive lobular	8	10.0	1	12.5	7	87.5	
HR status							.001
ER+/PR+	39	52.0	4	10.3	35	89.7	
ER+/PR-/Unknown	10	13.3	7	70.0	3	30.0	
ER-/unknown/PR+	2	2.7	1	50.0	1	50.0	
ER-/PR-	24	32.0	9	37.5	15	62.5	
Not documented	3	-					
HER2 status							.001
Negative	59	78.7	11	18.6	48	81.4	
Positive	16	21.3	10	62.5	6	37.5	
Not documented	3	-					
Drug Therapy							.001
AC → T	57	71.8	11	19.3	46	80.7	
AC → TH	9	11.5	8	88.9	1	11.1	
TCbH	7	9.0	2	28.6	5	71.4	
AC → TCb	3	3.8	1	33.3	2	66.7	
AC	2	3.6	0	0.0	2	100.0	
Treatment adherence							.018
Yes	60	76.9	21	35.0	39	65.0	
No	18	23.1	1	5.6	17	94.4	

Statistical tests for categorical factors were performed using the Pearson’s Χ^2^ test or Fisher’s exact test; tests for numerical factors were performed using a t-test

PR, progesterone receptor; ER, estrogen receptor; HER2, human epidermal growth factor receptor; pCR, pathologic complete response; A, anthracycline, T, paclitaxel or docetaxel, C, cyclophosphamide; Cb, carboplatin, H, trastuzumab

The overall pCR rate for the SpheroNEO study cohort was 28.2 % (22 out of 78). This rate is similar to other studies where the majority of patients were also treated with an anthracycline/taxane-based regimen [3, 42]. As expected, pCR rate was higher in younger patients, hormone receptor negative patients, as well as HER2-positive patients (Table 2). Treatment non-adherence defined as treatment discontinuation, dose-reduction, or change of treatment regime resulted in a lower pCR rate (*p* = .016).

### Characterization of the breast cancer spheroid model

Tumor spheroids were directly derived from the tissue samples without selecting any specific cell type [43]. Due to the heterogeneity of the tissue, the breast cancer spheroids varied between each patient in regard to cellular composition. The average size and compaction of the spheroids was dependent on the number of cells per spheroid and the cellular composition. Spheroid morphology was classified as compact, intermediate, and loose as previously published [40] and correlated with the patient characteristics. The spheroid morphology was not effected by age (*p* = .678), cT Stadium (*p* = .064), nodal status (*p* = .473), hormone receptor (*p* = .256) or HER2 status (*p* = .082), as well as Ki67 (p = .536). A lobular histology also did not have an impact on spheroid morphology, however only 8 cases were included in the main analysis. The only factor which was associated with significant differences in spheroid morphology was the grading of the tumor (*p* = .009). High grade tumors tended to form less compact spheroids while low grade tumors generated more compact spheroids.

### Predictive power of the breast cancer spheroid model for treatment outcome

**Fig. 2:**
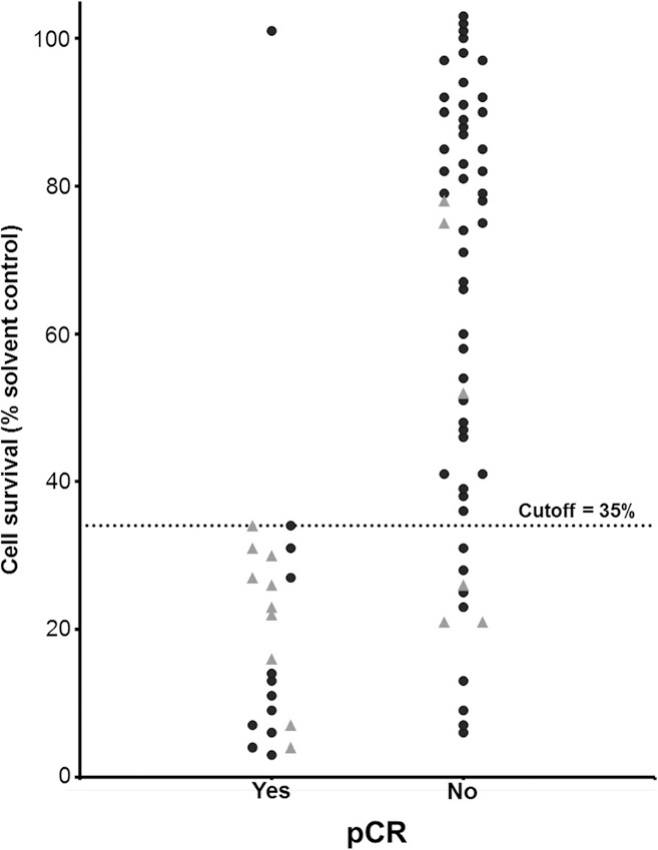
Dot histogram showing the mean cell survival of each tumor sample following cytostatic treatment in the breast cancer spheroid model. The results are grouped according to pCR. Each dot represents one patient; triangles represent patients treated with trastuzumab-based therapy. Proposed cutoff to predict pCR is shown at 35 % cell survival

A significant difference in cell survival *in vitro* was found in a comparison of patients achieving pCR versus those who did not (*p* = .001; Fig. 2). A mean cell survival of 21.8 % was found in the breast cancer spheroid model for patients with pCR versus 63.8 % in non-pCR patients. For trastuzumab-based regimen a mean of 21.9 % versus 45.4 % was seen in patients achieving or not achieving pCR respectively (*p* = .085).

Hormone-receptor negative tumor samples (43.03 %, *p* = .038) and HER2-positive tumor samples (30.7 %, *p* < .0001) showed lower cell survival rates than hormone-receptor-positive or HER2-negative tumor samples, respectively (Table 3).

**Table 3:** Correlation of cell survival with Clinical and Pathological Factors

			Cutoff				
	Mean		<35 %		≥35 %		
Characteristics	%	*P*	n	%	n	%	*P*
All Patients			32	41.0	46	59.0	
Age at diagnosis, years		.877					1.000
≤50	51.44		18	41.9	25	58.1	
>50	52.61		14	40.0	21	60.0	
Mean	-		51		51		.817
Range	-		21-76		25-78		
Tumor stage		.181					.603
cT1/T2	49.40		24	42.9	32	57.1	
cT3/4	60.66		7	33.3	14	66.7	
Nodal status		.813					1.000
cN+	50.41		17	40.5	25	59.5	
cN-	52.20		15	42.9	20	57.1	
Grading		.339					.486
G1/2	55.34		15	36.6	26	63.4	
G3	48.09		16	45.7	19	54.3	
Not documented			-		-		
Histologic type		.569					.439
Ductal invasive/other	51.25		30	42.9	40	57.1	
Lobular invasive	58.29		2	25.0	6	75.0	
HR status		.038					.042
ER+/PR+	62.49		10	25.6	29	74.4	
ER+/PR-/Unknown	36.10		7	70.0	3	30.0	
ER-/unknown/PR+	47.82		1	50.0	1	50.0	
ER-/PR-	43.03		12	50.0	12	50.0	
HER2 status		.000					<.0001
Negative	58.34		17	28.8	42	71.2	
Positive	30.70		13	81.3	3	18.8	

PR, progesterone receptor; ER, estrogen receptor; HER2, human epidermal growth factor receptor; pCR, pathologic complete response

**Fig. 3:**
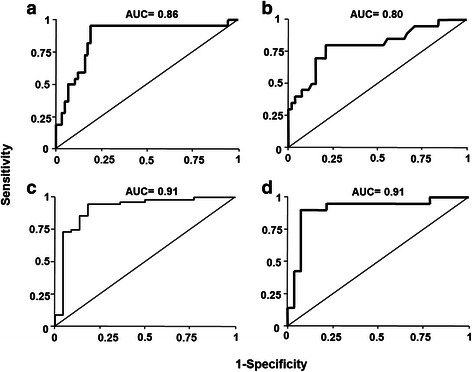
Receiver operator curves (ROC) displaying the sensitivity and specificity of (**a**) the breast cancer spheroid, (**b**) a multifactor model with baseline predictive factors (ER, PR, HER2, and age) impacting pCR, (**c**) model combining the breast cancer spheroid model, HER2-Status and type of therapy, and (**d**) breast cancer spheroid model combined with the factor of treatment adherence defined as treatment discontinuation, dose-reduction or change of treatment. The resulting area under the curve (AUC) is displayed at the top of each curve

The predictive power of the breast cancer spheroid model was analyzed in the context of established predictors. The c statistic (area under the ROC curve) for the treatment results in the breast cancer spheroid model was 0.86 (Fig. 3a), which was superior to classic risk factors. The AUC for the standard clinical factors significantly correlated with pCR in this cohort which were hormone-receptor status, HER2, and age resulted in an AUC of 0.80 (Fig. 3b). Simultaneous modelling of the effects of the spheroid model and clinical predictors in lasso regression models resulted in a slight increase of the AUC to 0.91 when HER2 and the type of therapy were considered, since patients receiving a trastuzumab-based therapy experienced higher pCR rates (Fig. 3c). Patients who did not receive the planned therapy had a lower rate of pCR (odds ratio = −2.94, *P* = .0126) The AUC from the model considering assay results and treatment adherence exhibited an AUC of 0.91 (Fig. 3d).

**Fig. 4:**
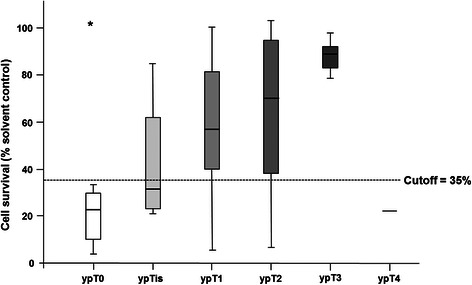
Box plot diagram showing mean cell survival in the breast cancer spheroid model after cytostatic treatment in comparison to the remaining tumor as determined by the pathological assessment of the surgical specimen after chemotherapy. Proposed cutoff of 35 % is represented by the dotted line. ypT0/ypN0 = 24.4 % (*n* = 19), ypTis =5.1 % (*n* = 4; ypN0 2 out of 3), ypT1 = 33.3 % (*n* = 26), ypT2 = 20.5 % (*n* = 16), ypT3 = 10.3 % (*n* = 8), ypT4 = 1.3 % (*n* = 1), not documented (*n* = 4), * represents an outlier

In addition to the pCR, correlation of cell survival with the ypT-stage revealed that the breast cancer spheroid model was significantly related to the gradual response as seen in the surgical specimen after chemotherapy (Fig. 4; Spearman’s rho = .311, *p* = .021).

A cutoff associated with pCR in the breast cancer spheroid model was detected at 35 % cell survival. This cutoff demonstrated a sensitivity of 95.5 % (21 out of 22) and a specificity of 80.4 % (45 out of 56).

Correlation of clinical and pathological data with the cutoff showed similar results in comparison to the reported correlations with mean cell survival (Table 3).

Odds ratios calculated with clinical and laboratory subgroups confirmed that HER2 status, treatment adherence, and cutoff in the breast cancer spheroid model significantly impact pCR (Table 4).

**Table 4:** Odds ratios for the clinical and laboratory variables impacting pCR in a multivariable model

Characteristics	Odds ratio	95 % CI	*P*
Cell survival cutoff (>35 %/≤ 35 %)	0.011	0.001 – 0.096	.0001
Treatment adherence (no/yes)	0.115	0.014 – 0.925	.0421
HR status (pos/neg)	0.513	0.180 – 1.465	.2123
HER2 status (neg/pos)	0.167	0.050 – 0.560	.0037
Age (>50/≤50 years)	0.501	0.177 – 1.417	.1505

## Discussion

The results of the SpheroNEO Study indicate that the *in vitro* breast cancer spheroid model correctly identified outcome for each treatment combination on an individual patient basis. Tumor spheroids reflect the cancer biology of the individual tumor more accurately compared to traditional 2-D cell assays. A tumor spheroid model, such as the one tested here, simulates the heterogeneity of cell types, cell-cell interactions, and the microenvironment of the patient tumor much more closely as compared to cell monolayer assays [44–46], in addition, through the 3-D structure a penetration barrier is formed allowing different concentrations of cytostatic agent to reach each tumor cell [47].

Cell-based chemosensitivity/chemoresistence assays have been tested since the early 1970s, and the results seemed initially promising [28, 32, 48, 49]. However, implementation of these assays into the clinical routine was not successful [32, 50, 51]. With an abundance of available drugs and targeted therapies today, the number of available treatment options for each patient is increasing, and there is a necessity for a preclinical model to stratify each patient to the optimal treatment option [52].

The aim of the present study was to introduce a pre-clinical *in vitro* assay to tailor the best possible anti-tumor treatment for breast cancer patients in the neoadjuvant setting. There are some limitations regarding laboratory methodology. The main difficulty was the minimal number of cells required for a reliable assay outcome. The methodology had previously been established using surgical specimen from colorectal carcinoma patients, which consistently yielded a greater number of isolated cells. Adapting this method to the much smaller breast cancer biopsies proved challenging, especially since the anatomy of the breast is much more diverse in its tissue components. This resulted in a wide range of total isolated cell number per sample weight.

A second factor that influenced the drop-out rate of patients was due to the timeline of the neoadjuvant treatment. At the time of biopsy for the initial histological confirmation no final decision regarding the chemotherapy regimen had been made for the respective breast cancer patient. This explains the considerable number of patients beginning a different neoadjuvant treatment as initially planned, receiving primary surgery with or without adjuvant treatment instead. The final treatment decision is made in collaboration between patient and physician, taking many clinical and non-oncologic factors into account. Treatment non-adherence was most frequent in older women, as well as in patients with one or multiple comorbidities.

*In vitro* treatment efficacy results comparing standard treatment combinations for all patients in this study recapitulate treatment options as outlined for various breast cancer subgroups in current guidelines. The high standard deviations of these results reflect the heterogeneity of the patient subgroups. A total of six patients were identified where more than one treatment combination could be tested. The results of the breast cancer spheroid model correctly identified the efficacy of the clinical treatment combination by demonstrating a significantly lower cell survival compared to other treatment combinations tested. Interestingly at least two or more treatment combinations proved equally effective *in vitro*, implying that a decision between approved anthracyclines or taxanes could be made according to each patient’s comorbidity or tolerability.

Furthermore, the breast cancer spheroid model showed a predictive response to trastuzumab-based therapy prior to histopathological confirmation of the HER2 status. In the tumor samples tested *in vitro* with trastuzumab-based therapy, a selective effect was seen. If the *in vitro* treatment with trastuzumab showed no additional benefit as compared to chemotherapy alone, the patients were histologically confirmed HER2 negative (*n* = 5).

## Conclusion

These preliminary analyses indicate that the breast cancer spheroid model is not only predictive but might also be selective in discerning ineffective from effective treatment options.

In order to validate the results from this explorative study an interventional randomized controlled confirmatory study is planned, follow-up data will also be analyzed. The cutoff as seen in this cohort of patients will be analyzed in regard to its validity and reliability in a larger cohort of patients.

## Additional file

Additional file 1:
**List of participating study sites and applicable ethics committees.**

## Abbreviations

AUC: Area under the curve
CI: Confidence interval
cps: Counts per second
HER2: Human epidermal growth factor 2
mTOR: Mammalian target of rapamycin
pCR: Pathologic complete response
ppc: Peak plasma concentration
ROC: Receiver operator curve
TNR: True negative rate
TPR: True positive rate
