# Supplementary material for: Prospective cohort study using the breast cancer spheroid model as a predictor for response to neoadjuvant therapy – the SpheroNEO study
Source: BMC Cancer. 2015 Jul 15;15:519. doi: 10.1186/s12885-015-1491-7 (PMC4501185; doi:10.1186/s12885-015-1491-7)
Supplement: Additional file 1: — List of participating study sites and applicable ethics committees. [file 12885_2015_1491_MOESM1_ESM.doc]

**List of participating study sites and applicable ethics committees**

| **Nr.** | **Study Site** | **Ethics Committee** | **written consent** |
| --- | --- | --- | --- |
| **01 (coordinating site)** | **University Hospital, LMU Munich Department of Gynecology and Obstetrics** | **Ethikkommission der Med. Fakultät der LMU München**  **Pettenkoferstrasse 8**  **80336 München** | yes |
| 02 | Frauenklinik des Klinikums Landshut GmbH  Robert-Koch-Str. 3  84034 Landshut | Ethik-Kommission der Bayrischen Landesärztekammer  Mühlbaurstraße 16  81677 München | yes |
| 03 | Chirurgische Klinik Dr. Rinecker  Am Isarkanal 30  81379 München | Ethik-Kommission der Bayrischen Landesärztekammer  Mühlbaurstraße 16  81677 München | yes |
| 04 | Brustzentrum Starnberg  Oßwaldstr. 1  82319 Starnberg | Ethik-Kommission der Bayrischen Landesärztekammer  Mühlbaurstraße 16  81677 München | yes |
| 05 | Frauenklinik und Poliklinik der Technischen Universität München, Klinikum Rechts der Isar  Ismaninger Str. 22  81675 München | Ethikkommission der Fakultät für Medizin der Technischen Universität München  Ismaninger Straße 22  81675 München | yes |
| 06 | Brustzentrum des Städtischen Klinikums München, Klinikum Harlaching  Sanatoriumsplatz 2  81545 München | Ethik-Kommission der Bayrischen Landesärztekammer  Mühlbaurstraße 16  81677 München | yes |
| 07 | Institut für Röntgendiagnostik  Klinikum rechts der Isar der TUM  Ismaninger Straße 22  81675 München | Ethikkommission der Fakultät für Medizin der Technischen Universität München  Ismaninger Straße 22  81675 München | yes |
| 08 | Klinikum St. Marien Amberg Mariahilfbergweg 7 92224 Amberg | Ethik-Kommission der Bayrischen Landesärztekammer  Mühlbaurstraße 16  81677 München | yes |
| 09 | Leopoldina-Krankenhaus der Stadt Schweinfurt gGmbH  Gustav-Adolf-Str. 8  97422 Schweinfurt | Ethik-Kommission bei der Medizinischen Fakultät der Universität Würzburg Institut für Pharmakologie und Toxikologie Versbacher Str. 9 97078 Würzburg | yes |
| 10 | Klinik für Frauenheilkunde im Klinikum Nürnberg  Prof.-Ernst-Nathan-Str. 1  90419 Nürnberg | Ethik-Kommission der Bayrischen Landesärztekammer  Mühlbaurstraße 16  81677 München | yes |
| 11 | Städtisches Klinikum Karlsruhe gGmbH  Moltkestr. 90  76133 Karlsruhe | Ethik-Kommission bei der Landesärztekammer Baden-Württemberg  Jahnstraße 40  70597 Stuttgart | yes |
| 12 | Interdisziplinäres Brustzentrum der Frankfurter Diaknonie-Kliniken am Markus-Krankenhaus  Wilhelm-Epstein-Straße 2 60431 Frankfurt am Main | Ethik-Kommission der Landesärztekammer  in Hessem  Im Vogelsang 3  60488 Frankfurt | yes |
| 13 | Klinik für Gynäkologie und Geburtshilfe  Marienhospital Bottrop gGmbH  Josef-Albers-Str. 70  46236 Bottrop | Ethik-Kommission der Ärztekammer  Westfalen-Lippe  Gartenstraße 210-214  48147 Münster | yes |
| 14 | Evangelische Kliniken Gelsenkirchen GmbH  Klinik für Senologie  Munckelstr. 27  45879 Gelsenkirchen | Ethik-Kommission der Ärztekammer  Westfalen-Lippe  Gartenstraße 210-214  48147 Münster | yes |
